# Multi-Objective Optimization of a Long-Stroke Moving-Iron Proportional Solenoid Actuator

**DOI:** 10.3390/mi15010058

**Published:** 2023-12-27

**Authors:** Peng Liu, Yuwen Ouyang, Wenwen Quan

**Affiliations:** College of Automotive and Mechanical Engineering, Changsha University of Science & Technology, Changsha 410114, China

**Keywords:** solenoid actuator, correlation analysis, main effect analysis, multi-objective optimization, multi-criteria decision-making

## Abstract

In this study, the performance of a long-stroke moving-iron proportional solenoid actuator (MPSA) was improved by combining numerical simulations and experiments. A finite element model of the MPSA was developed; its maximum and mean relative absolute errors of electromagnetic force were 4.3% and 2.3%, respectively, under typical work conditions. Seven design parameters including the cone angle, cone length, depth of the inner hole of the coil skeleton, cone width of the armature, inner cone diameter, and initial position of the moving-iron core were selected for developing the model, and the coefficient of the variation in electromagnetic force, nominal acceleration, 95% of the maximum stable output electromagnetic force, and corresponding response time were used as the performance indicators. The constraint relation between each performance indicator and the influence of each design parameter on the performance indicators were revealed using the uniform Latin hypercube experiment design, correlation analysis, and the main effect analysis method. A multi-objective optimization mathematical model of the MPSA was developed by combining traditional surrogate and machine learning models. The Pareto solution set was obtained using the nondominated sorting genetic algorithm II (NSGA-II), and three decision schemes with different attitudes were determined using the Hurwicz multi-criteria decision-making method. The results showed that a strong contradiction exists among the 95% of the maximum stable output electromagnetic force and its corresponding response time and the coefficient of the variation in electromagnetic force. The cone angle considerably influenced the performance indicators. Compared with the initial design, the coefficient of the variation in electromagnetic force was reduced by 54.08% for the positive decision, the corresponding response time was shortened by 15.65% for the critical decision, and the corresponding acceleration was enhanced by 10.32% for the passive decision. Thus, the overall performance of the long-stroke MPSA effectively improved.

## 1. Introduction

A moving-iron proportional solenoid actuator (MPSA) is an electromechanical conversion device vital for automatic control systems and is widely used in several fields, such as hydraulic systems [1,2,3], common rail systems with direct injection equipment in diesel engines [4], hydraulic braking actuators of automobile antilock braking systems (ABSs) [5], automatic transmission pressure control devices [6], and incremental sheet-metal-forming tools [7], owing to its simple structure, large driving force, and low price [8,9]. The electromagnetic force characteristics and dynamic response speed of the MPSA influence the driving ability and control accuracy of a control system [10,11,12]. Thus, their overall performance must be improved.

The design and optimization of the MPSA have been extensively researched. Bayat et al. [9] analyzed the influence of the structural parameters of a pole shoe and various soft magnetic materials with different B–H curves, current density, and air gap sizes on the constant force characteristics of the MPSA. Yun et al. [4] studied the effects of the gap between the plunger and control cone and the length and width of the control cone on the constant force characteristics of the MPSA for common rail systems in diesel engines. They optimized the shape of the control cone using the finite element method. Arakawa et al. [11] strengthened the density and linearity of the electromagnetic force of the MPSA for a variable valve timing system using the optimization software iSIGHT. Yamada et al. [13] improved the constant force characteristics of the MPSA by adjusting the stator and mover shapes or changing the magnetic material type. They found that permendur and modified stator and mover shapes reduced the constant thrust range but increased the MPSA thrust. Xie et al. [14] designed a high-speed proportional solenoid and performed static and dynamic analyses using different electromagnetic materials, structural parameters, and critical variables. They found that the material of the solenoid core and dynamic performance are not highly correlated. Yuan et al. [15] studied the pole shoe structure of a flat-type proportional solenoid actuator and analyzed the influence of six magnetic materials and geometric parameters on the constant force characteristics in detail. Yun et al. [16,17] analyzed the characteristics of the force of attraction of the electromagnetic proportional solenoid actuator in pressure control valves by varying the dither-like duty cycle signal of pulse-width modulation (PWM) and optimized the structural parameters of the control cone using an optimization algorithm. Yu et al. [18] constructed and verified a 3D finite element model of proportional solenoids and showed via parameter sensitivity analysis that some key shape parameters considerably influence the electromagnetic force. They also adjusted and optimized their model. Wang et al. [19,20] optimized the structural parameters of the pole shoe and moving core of the MPSA by combining the 2D finite element model and multi-objective genetic algorithm.

These studies provided a solid theoretical foundation for the design and development of high-performance MPSAs. However, they focused on analyzing and optimizing individual performance or isolated performance targets and neglected the interdependencies between them, possibly resulting in low MPSA performance. Therefore, the coupling relation between various performance indicators and parameters must be quantified and clarified to enhance MPSA performance. Moreover, the functional relation between design parameters and optimization objectives must be established for efficient and accurate multi-objective optimization and MPSA-performance enhancement.

Herein, a long-stroke MPSA with an effective stroke of 10 mm was analyzed using numerical simulation, correlation analysis, and main effect analysis in combination, which revealed the coupling relation between various performance targets. Moreover, the degree of each design parameter and each design parameter’s influence on these performance indicators were determined. Traditional surrogate models and machine learning methods were integrated to establish a multi-objective optimization mathematical model with hybrid surrogate models for the long-stroke MPSA. The optimization solution was then determined using the nondominated sorting genetic algorithm II (NSGA-II) and multi-criteria decision-making. The results of this study provide a basis for the optimization design of proportional solenoid actuators.

## 2. Structure and Principle

**Figure 1:**
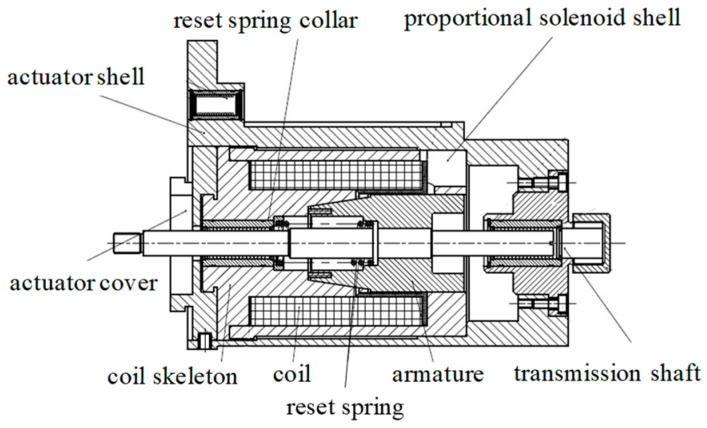
Structural representation of the moving-iron proportional solenoid actuator (MPSA).

Figure 1 schematizes the long-stroke MPSA, which mainly comprises an actuator shell, a proportional solenoid shell, an armature, a coil skeleton, a coil, a reset spring, a reset spring collar, a transmission shaft, and an actuator cover. When the coil is electrified, the proportional solenoid shell, armature, and coil skeleton are magnetized, generating an electromagnetic force between the armature and coil skeleton. When the driving current gradually increases and the electromagnetic force exceeds the spring preload force, the armature moves toward the coil skeleton and drives the transmission shaft, generating an electromagnetic force and causing a displacement in the controlled object. When the driving current decreases, the electromagnetic force decreases, the transmission shaft and armature return under the action of the reset spring, and the displacement of the controlled object decreases. Due to the proportional relation between the electromagnetic force and driving current, the output electromagnetic force of the MPSA and displacement of the controlled object can be precisely controlled by adjusting the driving current signal of the coil.

## 3. Optimization Problem and Method

### 3.1. Optimization Problem

#### 3.1.1. Performance Index

To enhance the control accuracy, system response speed, and driving capability of the controlled object, the MPSA must output substantial electromagnetic force and have favorable horizontal force characteristics and high dynamic response. Therefore, the coefficient of the variation in electromagnetic force *CV*(*i_n_*), 95% of the maximum stable output electromagnetic force *F*_0.95_ (hereinafter, output electromagnetic force *F*_0.95_), corresponding response time *t*_0.95_, and nominal acceleration *a* are introduced as performance indicators herein. *CV*(*i_n_*) reflects the horizontal force characteristics of the MPSA, while *F*_0.95_ indicates the strength of its output electromagnetic force. *t*_0.95_ and nominal acceleration *a* depict the dynamic response characteristics of the MPSA.

(1) Coefficient of the variation in electromagnetic force *CV*(*i_n_*): Based on the working range of the driving current, *CV*(*i_n_*) is defined in Equation (1) for a typical driving current to reflect the horizontal force characteristics of the MPSA under different driving current conditions. *CV*(*i_n_*) ≈ 0 indicates better horizontal force characteristics, whereas a larger *CV*(*i_n_*) indicates poor horizontal force characteristics.

(1) $$\left\{\begin{array}{l} CV\left(i_{n}\right)=\frac{F{\left(i_{n}\right)}_{s}}{F{\left(i_{n}\right)}_{a}}\;\;\;i_{n}\in \left[2,3,4\right]\\ F{\left(i_{n}\right)}_{a}=\frac{\sum_{m=1}^{f+1}F\left(i_{n},\;x_{m}\right)}{f+1}\;\;x_{m}\in \left[5,15\right]\\ F{\left(i_{n}\right)}_{s}=\sqrt{\frac{\sum_{m=1}^{f+1}{\left[F\left(i_{n},\;x_{m}\right)-F{\left(i_{n}\right)}_{a}\right]}^{2}}{f+1}} \end{array}\right.$$

where *i_n_* is the typical driving current; *x_m_* is the working stroke; *f* is the number of equal divisions for the total working stroke, which is 10; *CV*(*i_n_*) is the coefficient of the variation in electromagnetic force when the driving current is *i_n_*; *F*(*i_n_*)_a_ is the average output electromagnetic force within the working stroke range when the driving current is *i_n_*; *F*(*i_n_*)_s_ is the standard deviation of the output electromagnetic force within the working stroke range when the driving current is *i_n_*; and *F*(*i_n_*, *x_m_*) is the output electromagnetic force when the driving current is *i_n_* and the working stroke is *x_m_*.

**Figure 2:**
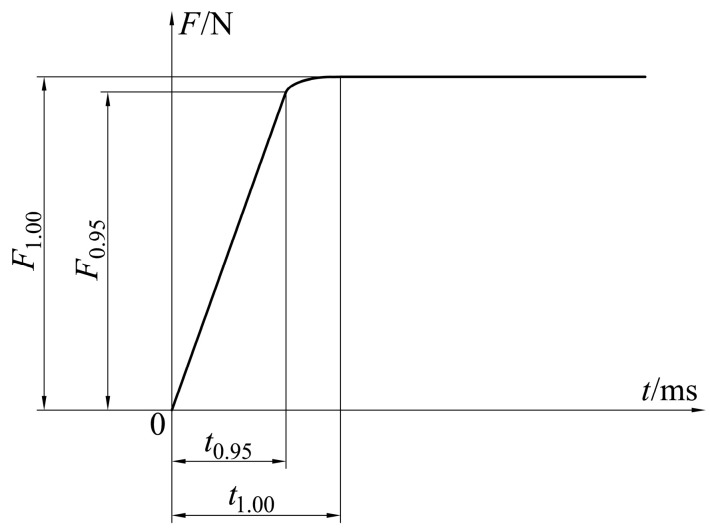
Output electromagnetic force transient response characteristics for the MPSA.

(2) Output electromagnetic force *F*_0.95_: When the armature is in the middle working stroke of 10 mm and under the step driving voltage signal of 24 V, 95% of its maximum stable output electromagnetic force *F*_1.00_ produced by the actuator is called the output electromagnetic force *F*_0.95_ (Figure 2). A larger output electromagnetic force *F*_0.95_ indicates a stronger driving force of the MPSA.
(3) Response time *t*_0.95_: When the armature is in the middle working stroke, the time required for the actuator to reach 95% of its maximum stable output electromagnetic force *F*_1.00_ under the step drive voltage signal is called the response time *t*_0.95_ (Figure 2). A smaller response time *t*_0.95_ indicates a faster response speed by the MPSA.
(4) Nominal acceleration a: The ratio of the output electromagnetic force *F*_0.95_ to the mass of the moving parts of the MPSA is defined as the nominal acceleration *a* (Equation (2)). A larger nominal acceleration a indicates that MPSA has good acceleration performance and a high output electromagnetic force density.

(2) $$\left\{\begin{array}{l} a=\frac{F_{0.95}}{m}\\ m=0.41+7.87V \end{array}\right.$$

where *m* is the mass of moving parts of the MPSA, and *V* is the volume of armature.

#### 3.1.2. Design Parameters

**Figure 3:**
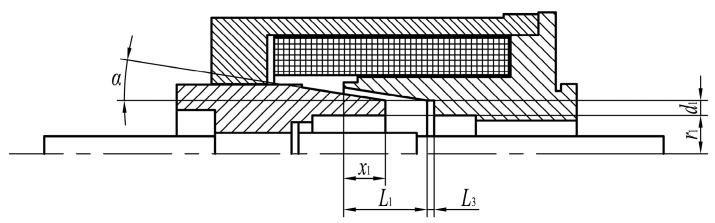
Schematic of the optimized design parameters.

Magnetic circuit analysis reveals that the MPSA performance is mainly affected by the shape of the pole shoe and the size of the working air gap. Thus, seven optimization design parameters are considered herein, including five pole shoe parameters (cone angle *α*, cone length *L*_1_, inner hole depth of coil skeleton *L*_3_, cone width *d*_1_, and cone inner diameter *r*_1_), air gap control parameters (armature initial position *x*_0_), and coil turns *N*. The positions of these parameters and their values are shown in Figure 3 and Table 1, respectively.

### 3.2. Optimization Method

**Figure 4:**
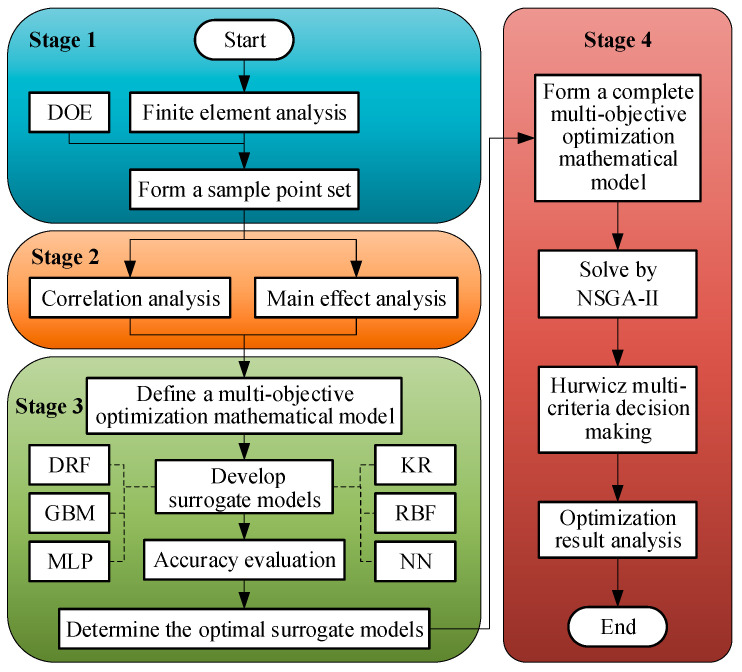
Implementation process.

Herein, the performance of MPSA is studied and optimized by combining various techniques and methods such as finite element analysis, experimental design, correlation analysis, main effect analysis, surrogate model, genetic algorithm, and multi-criteria decision-making. The process comprises the following four stages (Figure 4).

Stage 1: The finite element model of the long-stroke MPSA is developed using professional electromagnetic simulation software. Then, 100 groups of sample points are generated using the uniform Latin hypercube design of experiment (DOE) [21,22] and imported into the finite element model for numerical analysis. The response values of electromagnetic force *F*(*i_n_*, *x_m_*), response time *t*_0.95_, and output electromagnetic force *F*_0.95_ at each discrete operating condition point are directly obtained. Further, the response values of *CV*(*i_n_*) are calculated using Equation (1). The sample point set is then generated for subsequent analysis and optimization.

Stage 2: Based on the sample point set generated in stage 1, the coupling relation between various performance indicators is determined using correlation analysis methods, and the influence degree of each design parameter on the performance indicators is determined via main effect analysis.

Stage 3: The multi-objective optimization mathematical model is defined. Then, the functional relation between design parameters and performance objectives and constraints is determined based on the sample point set data by combining traditional surrogate models such as Kriging (KR) [23], radial basis function (RBF) [24], and artificial neural network (ANN) [25] and machine learning methods such as distributed random forest (DRF) [26,27], gradient boosting machine (GBM) [28,29], and multilayer perceptron (MLP) [30]. Additionally, the optimal surrogate model scheme and model are developed.

Stage 4: The complete multi-objective optimization mathematical model of the long-stroke MPSA is established based on the optimal surrogate model. The Pareto solution set is obtained using NSGA-II. Then, the optimal solution is determined using Hurwicz multi-criteria decision-making method [31] and validated and analyzed using the finite element method.

## 4. Finite Element Model

**Figure 5:**
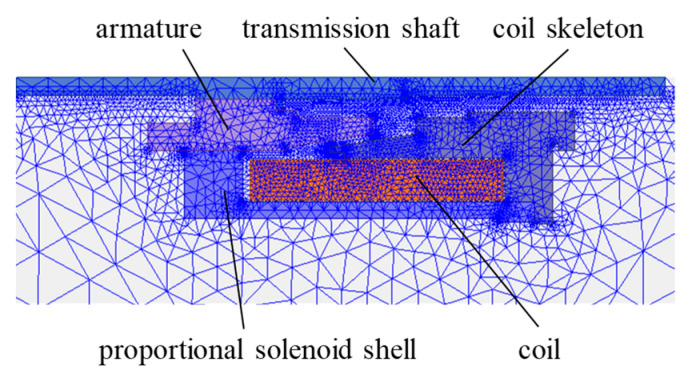
Finite element model of the long-stroke MPSA.

The professional electromagnetic simulation software, ANSYS Maxwell 2020 R1, is used herein to develop the finite element models of the long-stroke MPSA, encompassing both the magnetostatic and transient magnetic models. The magnetostatic model is employed to obtain the electromagnetic force *F*(*i_n_*, *x_m_*), while the transient magnetic model is utilized to ascertain the response time *t*_0.95_ and the output electromagnetic force *F*_0.95_. As the long-stroke MPSA has a closed magnetic circuit, the magnetic lines of force are mainly concentrated in the magnetic circuit composed of soft magnetic materials, such as the proportional solenoid shell, armature, coil skeleton, and transmission shaft; thus, the magnetic flux leakage is small. Therefore, these components are mainly considered during finite element modeling. As these components have a symmetric geometric structure, the two-dimensional axisymmetric modeling method is used to improve the solving speed. The magnetostatic model of the long-stroke MPSA adopts the adaptive meshing method, and its convergence criterion is the set energy error or the maximum number of iterations. Its excitation is determined by the ampere-turns, which equals the product of the coil turns and the driving current. The transient magnetic model of the long-stroke MPSA directly adopts the convergent mesh from the magnetostatic model. A 24 V voltage is applied to the model, and the resistance of the loop is determined by the number of coil turns. Figure 5 shows the simplified finite element model and the mesh after solving the convergence.

**Figure 6:**
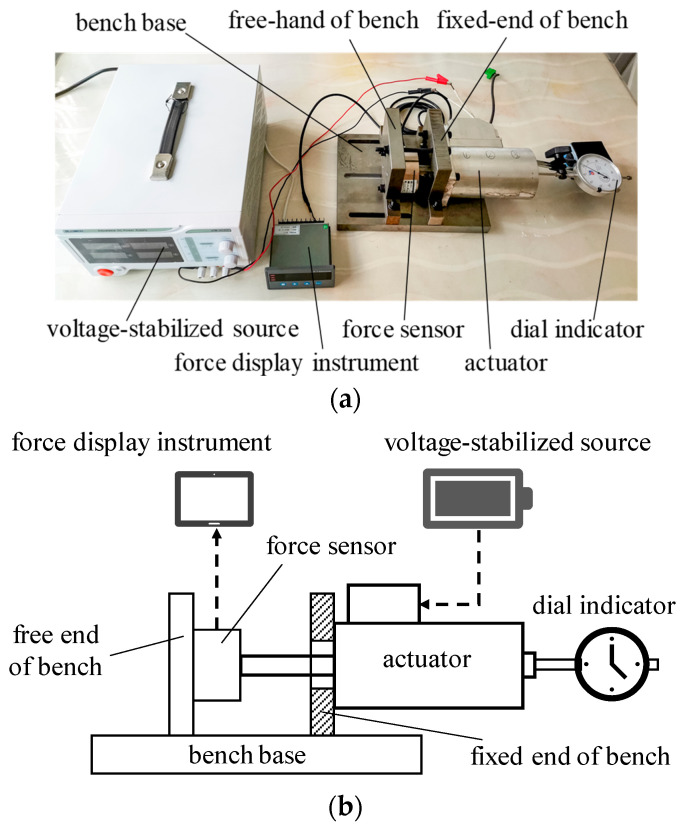
Test device for determining the electromagnetic force of the MPSA: (**a**) photo image of test device; (**b**) schematic diagram of test device.

The magnetic field must be accurately predicted for the finite element modeling of the long-stroke MPSA. The electromagnetic force reflects the action of the magnetic field. Therefore, the accuracy of the finite element models is verified by comparing the experimentally determined electromagnetic force with the simulation results. Figure 6 shows the electromagnetic force test platform of the long-stroke MPSA, which is mainly composed of a test bench, force sensor, voltage-stabilized source, force display instrument, and dial indicator. The actuator and force sensor are fastened on the fixed end and free hand of the bench, respectively, during the test; the transmission shaft of the actuator is located in line with the force sensor. The actuator stroke is adjusted using the free hand and locked when it moves to the designated location (measured by a caliper). When the constant drive current passes through the actuator coil, (the drive current is adjusted by a voltage-stabilized source and then output), the armature overcomes the preload of the reset spring due to the electromagnetic force and drives the transmission shaft to the designated location (the actual stroke of the actuator’s transmission shaft is recorded by a dial indicator). Subsequently, under the action of electromagnetic force, the transmission shaft pushes the force sensor to produce a weak voltage signal. This signal is sent to a force display instrument for amplification, yielding the net electromagnetic force (the difference between the electromagnetic force and spring force). With known spring preload and stiffness values, the electromagnetic force of the actuator can be calculated by Equation (3).

(3) $$F(i_{n},x_{m})=F_{\mathrm{net}}+F_{0}+kx_{m}$$

where *F*_net_ is the net electromagnetic force; *F*_0_ is the spring preload; and *k* is the spring stiffness.

**Figure 7:**
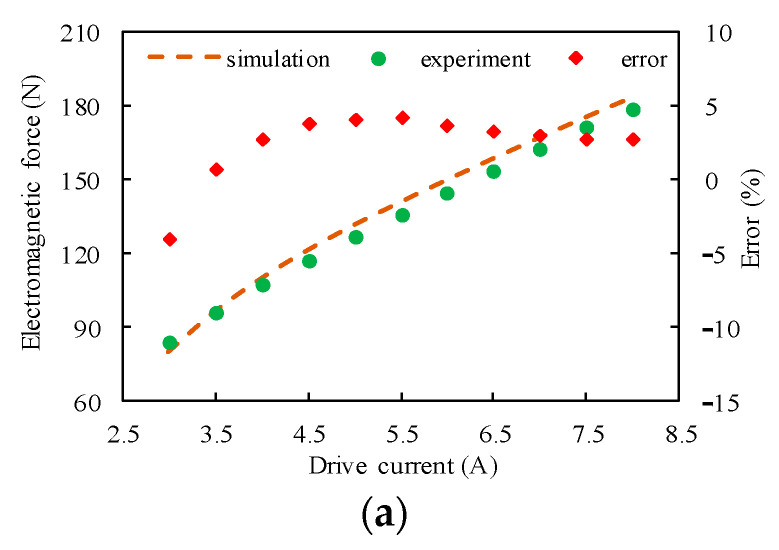

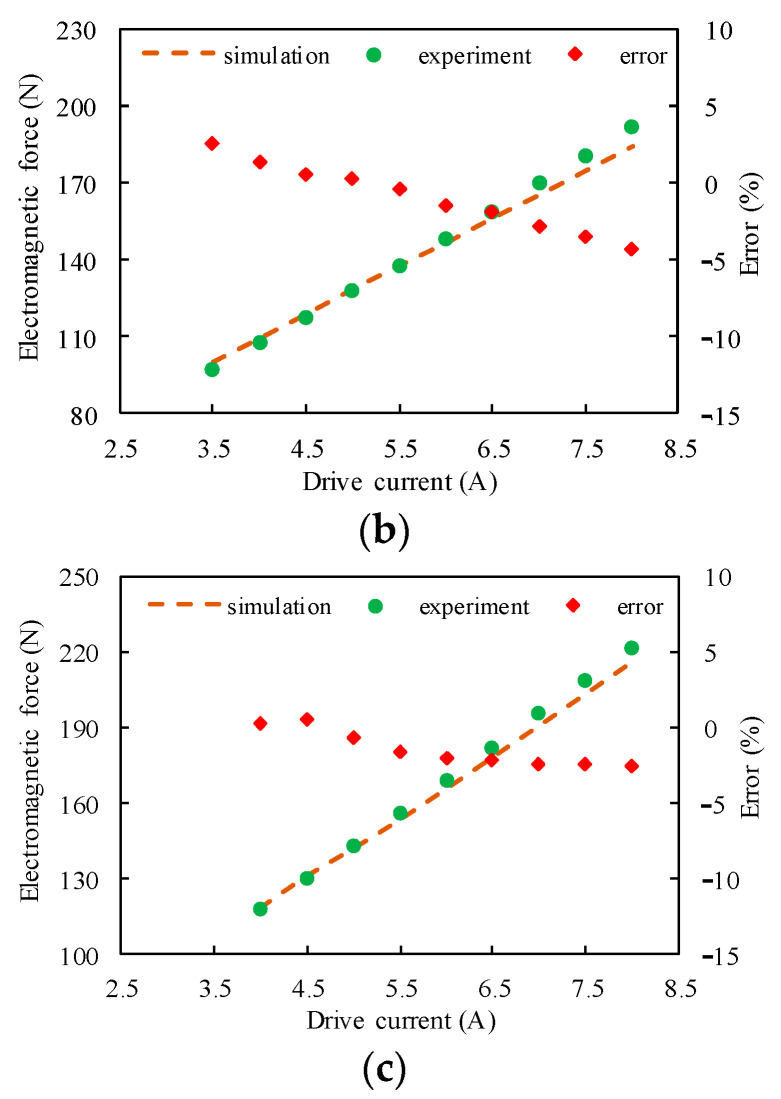
Comparison of the simulation and experimental results, (**a**–**c**) with working strokes of 5, 10, and 15 mm, respectively.

Figure 7 compares the experimentally determined solenoid force and the FEM results after calibration under a typical working stroke and driving current. The simulation results agree well with the experimental results; the maximum relative absolute error of the solenoid force under these working conditions is 4.3%, and the average relative absolute error is 2.3%.

## 5. Results and Discussion

### 5.1. Correlation Analysis

Correlation analysis is used to determine the degree and relation between two or more parameters and expressed as the Pearson correlation coefficient (hereinafter, correlation coefficient), as shown in Equation (4).

(4) $$R=\frac{\mathrm{cov}\left(X,Y\right)}{\sigma_{X}\sigma_{Y}}$$

where *R* is the correlation coefficient; cov(*X*, *Y*) is the covariance of variable *X* and variable *Y*; *σ_X_* and *σ_Y_* are the standard deviations of variable *X* and variable *Y*, respectively.

The correlation coefficient ranges from −1 to +1; Table 2 shows the correlation strength. The larger the absolute value of the correlation coefficient is, and the higher the correlation strength is, the more significant the influence of one variable on the other is. The positive and negative signs of the correlation coefficient indicate the direction of influence [32].

**Figure 8:**
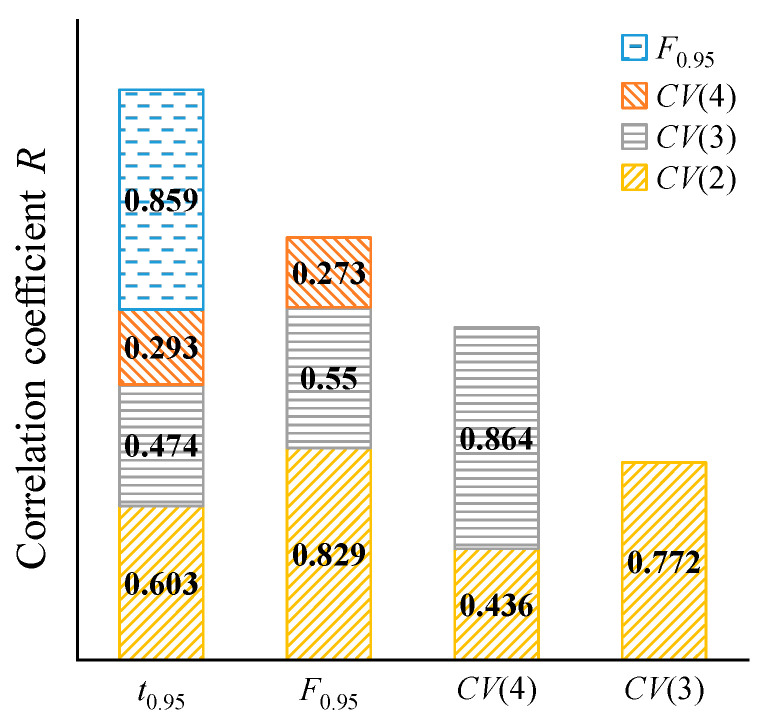
Correlations among the performance indicators.

Based on the 100 sample points generated in stage 1 and by Equation (3), the correlation coefficients between the performance indicators are obtained, as shown in Figure 8. They indicate a certain coupling or contradictory relation amongst these indicators.

(1) The correlation coefficient of response time *t*_0.95_ and output electromagnetic force *F*_0.95_ is 0.859, indicating a strong positive correlation between them. Thus, the larger the output electromagnetic force of the MPSA is, the longer the required response time is. In practice, a larger electromagnetic force can presumably be obtained in the shortest possible response time. Therefore, the response time *t*_0.95_ and output electromagnetic force *F*_0.95_ strongly contradict each other.

The response time *t*_0.95_ and *CV*(*i_n_*) are positively correlated, but this correlation gradually decreases with increasing driving current. This indicates that the faster the response time of the output electromagnetic force of the MPSA is, the better the horizontal characteristics are. In reality, the response time of the electromagnetic force must be faster, while maintaining a good horizontal force characteristic. This expectation coincides with the direction of optimization. However, as the driving current increases, the correlation coefficient decreases from 0.603 to 0.293 and changes from a strong correlation to a weak correlation. This weakens the degree of codirection optimization and increases the difficulty of optimization.

(2) A positive correlation exists between the output electromagnetic force *F*_0.95_ and *CV*(*i_n_*), indicating that both these indicators proportionally increase. Thus, optimizing one indicator inevitably degrades the other. However, with an increase in the driving current, the correlation coefficient decreases from 0.829 to 0.273 and changes from a strong correlation to a weak correlation. So the contradiction weakens.
(3) The correlation coefficient between different *CV*(*i_n_*) ranges from 0.436 to 0.864, showing a significant positive correlation. This indicates that the horizontal force characteristics under each driving current have different degrees of coupling relation; they simultaneously increase and decrease, which is conducive to improving the horizontal force characteristic for each driving current.

### 5.2. Main Effect Analysis

#### 5.2.1. Main Effect on the Horizontal Force Characteristics

**Figure 9:**
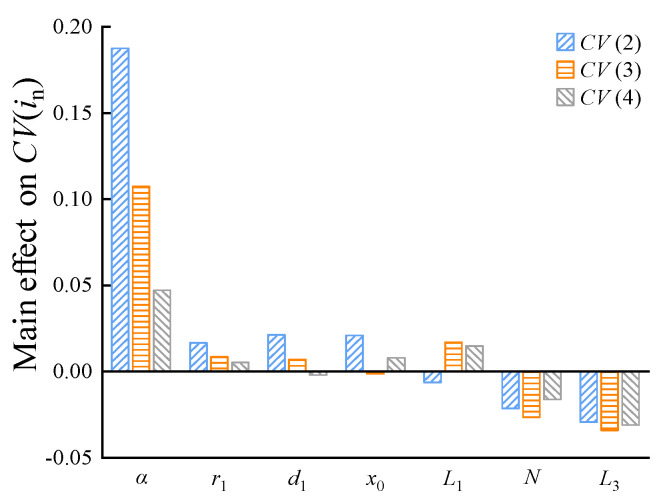
Main effect of the design parameters on the coefficient of the variation in electromagnetic force.

Figure 9 shows the main effect of the design parameters on *CV*(*i_n_*). As can be seen from Figure 9, the main effect of these parameters on *CV*(*i_n_*) has a positive and a negative. The positive means the increase in *CV*(*i_n_*) occurs with the increase in the parameter, and the negative means the decrease in *CV*(*i_n_*) occurs with the increase in the parameter.

In general, *α* has the most significant positive effect on *CV*(*i_n_*), whereas *r*_1_, *d*_1_, *x*_0_, and *L*_1_ have relatively little positive effect on *CV*(*i_n_*). *N* and *L*_3_ have obvious negative effects on *CV*(*i_n_*). Moreover, the influence of *α*, *r*_1_, and *d*_1_ on *CV*(*i_n_*) weakens with an increase in the driving current. This is primarily because the greater the driving current is, the more the magnetic field of the armature cone tends to saturation and the sensitivity of *α* and *r*_1_ to the magnetic field decreases. In addition, as the driving current increases, the influence of *d*_1_, *x*_0_, and *L*_1_ on *CV*(*i_n_*) switches between positive and negative effects, and the influence of *N* and *L*_3_ on *CV*(*i_n_*) exhibits a nonlinear change law. This is because the driving current increases and the magnetic field nonlinearly changes; thus, the influence of these aforementioned parameters on the electromagnetic force shows different variation laws under different magnetic field states.

#### 5.2.2. Main Effect on the Output Electromagnetic Force and Its Response Time

**Figure 10:**
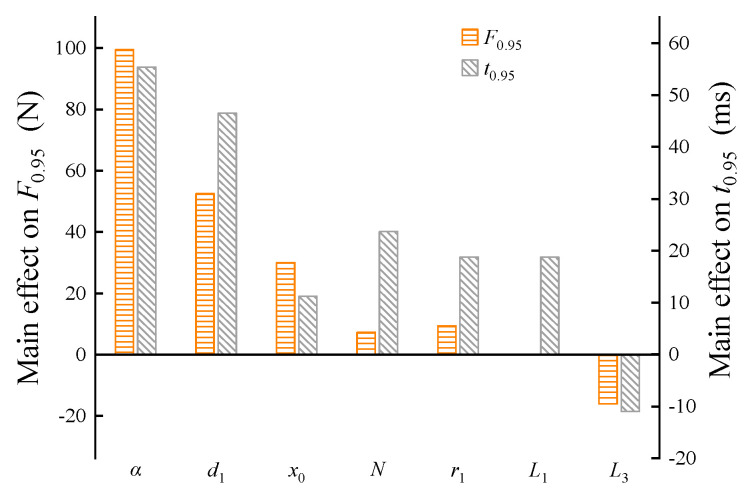
Main effect of design parameters on the output electromagnetic force and response time.

Figure 10 shows the main effect of the design parameters on the output electromagnetic force and its response time. As can be seen from Figure 10, with the exception of parameter *L*_3_, the main effects of the remaining parameters on the output electromagnetic force and its response time are positive.

Additionally, the main effects of *α* and *d*_1_ on the output electromagnetic force *F*_0.95_ and response time *t*_0.95_ are strong, whereas those of the other parameters are relatively weak, but the influence directions are the same. Thus, each design parameter must be considered as a compromise during the optimization process, which is a strong nonlinear optimization problem. In particular, the main effect of α on the output electromagnetic force *F*_0.95_ and response time *t*_0.95_ is relatively strong and close; thus, an increase in a target inevitably decreases the other target. Therefore, comprehensive optimization must be performed by the coordinated adjustment of other parameters with large differences in their main effects.

### 5.3. Multi-Objective Optimization

#### 5.3.1. Development of the Multi-Objective Optimization Mathematical Model

To improve the dynamic response speed and the horizontal force characteristics of the long-stroke MPSA and ensure that the driving ability and other performance indicators are not deteriorated, a multi-objective optimization mathematical model is developed, as shown in Equation (5). Minimizing the response time *t*_0.95_ and the average coefficient of the variation in electromagnetic force *CV*(*i_n_*)_a_, as well as maximizing the nominal acceleration *a*, are the objective functions.

(5) $$\left\{\begin{array}{l} \min\;t_{0.95}(z)\;\mathrm{and}\;CV(z)\\ \max\;a(z)\\ S.t.\;CV(z)=CV{\left(i_{n}\right)}_{a}\\ \;\;z=[\alpha ,L_{1},L_{3},N,d_{1},r_{1},x_{0}]\\ \;\;z_{i}^{l}\leq z_{i}\leq z_{i}^{u}\;(i=1,2,\ldots ,7)\\ \;\;t_{0.95}\leq t_{0.95}^{*}\\ \;\;F_{0.95}\geq F_{0.95}^{*}\\ \;\;CV{\left(i_{n}\right)}_{a}\leq CV{\left(i_{n}\right)}_{a}^{*}\\ \;\;F{\left(3\right)}_{a}\geq F{\left(3\right)}_{a}^{*} \end{array}\right.$$

where *CV*(*i_n_*)_a_ is the average coefficient of the variation in electromagnetic force. t^*^_0.95_, F^*^_0.95_, and *CV*(*i_n_*)^*^_a_ are the response time *t*_0.95_, output electromagnetic force *F*_0.95_, and average coefficient of the variation in electromagnetic force of the initial MPSA, which refers to the non-optimized MPSA, respectively. *F*(3)^*^_a_ is the average electromagnetic force of the initial MPSA under different strokes when the driving current is 3A. $z_{i}^{l}$ and $z_{i}^{u}$ are the lower and upper limits of the design parameter shown in Table 1, respectively.

#### 5.3.2. Development of the Optimal Surrogate Model

In the multi-objective optimization mathematical model (Equation (4)), the functional relations among the performance objectives (the response time *t*_0.95_, *CV*(*i_n_*)_a_, and *a*), the constrained electromagnetic force *F*(3)_a_, and the design parameters are unknown. Therefore, the surrogate models are used to replace the actual functional relations to efficiently predict the response values. As *CV*(*i_n_*)_a_ involves the nonlinear operation of standard deviation, a high-precision surrogate model for the electromagnetic force *F*(*i_n_*, *x_m_*) at each operating point is first constructed. Then, its mean value is obtained based on *CV*(*i_n_*). Similarly, a high-precision surrogate model for the output electromagnetic force *F*_0.95_ is first constructed and combined with the accurate expression of the moving part mass of the MPSA to develop the surrogate model for *a*. This can considerably reduce the difficulty of constructing the corresponding surrogate model and improve prediction accuracy.

Therefore, the 100 groups of sample points generated in stage 1 are divided, with 80% and 20% as the training and validation sample point sets, respectively. To reduce the influence of sample random segmentation on the surrogate model accuracy, 20 random segmentation tests are performed. The accuracy of the surrogate models is measured by the mean coefficient of determination *R*^2^.

**Figure 11:**
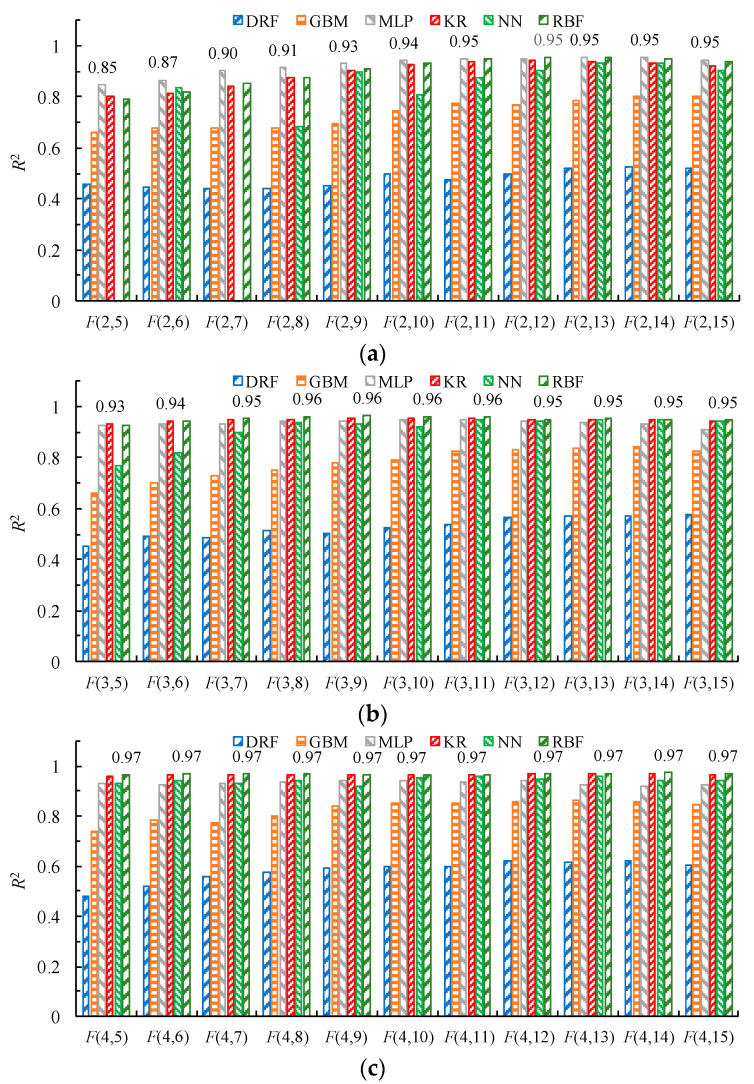
Mean coefficient of determination for the output electromagnetic force surrogate models at operating points, (**a**–**c**) with driving currents of 2, 3, and 4 A, respectively.

Figure 11 shows the *R*^2^ of surrogate models for the electromagnetic force *F*(*i_n_*, *x_m_*) at each operating point. Under a low current (2 A), the overall accuracy of the MLP surrogate models is relatively high. Under higher currents (3 A and 4 A), the overall accuracy of the RBF surrogate models is relatively high. Therefore, the MLP and RBF models are comprehensively selected as the main surrogate models. For operating points with poor prediction performance of the electromagnetic force, other high-accuracy surrogate models are used. Finally, the optimal surrogate model of the electromagnetic force at each operating point is shown in Table 3.

**Figure 12:**
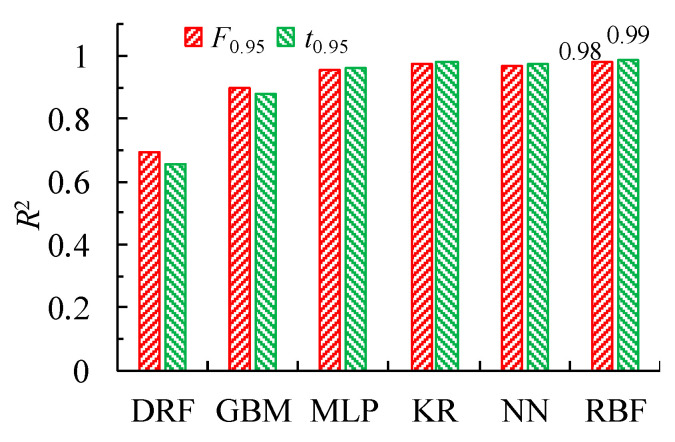
Mean coefficient of determination for the surrogate models of the output electromagnetic force *F*_0.95_ and response time *t*_0.95_.

Figure 12 shows the *R*^2^ of the surrogate models for the output electromagnetic force *F*_0.95_ and response time *t*_0.95_. The *R*^2^ of the MLP, KR, NN, and RBF models is >0.95, indicating a good prediction performance. The RBF model has the highest accuracy and can, therefore, be chosen as the optimal surrogate model for the output electromagnetic force *F*_0.95_ and response time *t*_0.95_.

#### 5.3.3. Solution and Analysis of the Multi-Objective Optimization Mathematical Model

**Figure 13:**
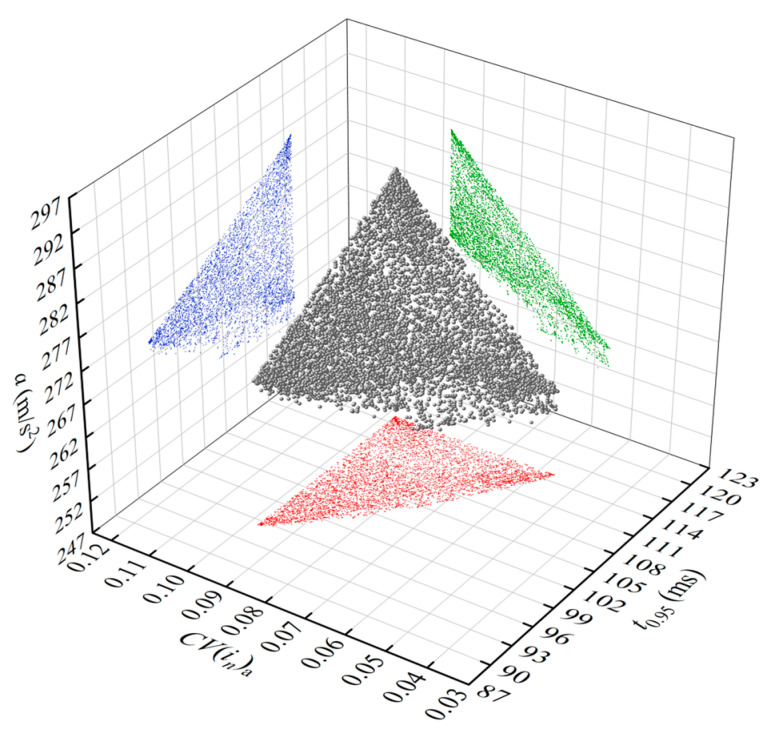
The Pareto solution set for multi-objective optimization.

NSGA-II is used to solve the multi-objective optimization mathematical model. The initial population, evolution generation, crossover probability, and mutation probability are set to 200, 200, 0.9, and 0.5, respectively. The Pareto solution set is obtained, as shown in Figure 13, wherein a certain contradiction can be observed among the optimization solutions of *CV*(*i_n_*)_a_, *a*, and the response time *t*_0.95_. These indicators cannot simultaneously reach their optimum values. Therefore, the Hurwicz multi-criteria decision is used to determine the optimal solution.

The Hurwicz criterion, or the criterion of realism, is one of the commonly used methods for decision-making under uncertainty and represents a compromise between the optimistic and pessimistic approaches [33,34]. The decision-maker expresses their degree of optimism by setting the Hurwicz weight *λ* (the coefficient of optimism or alpha) in the range of 0–1, where 0 indicates total optimism and, therefore, corresponds to the maximax criterion, and 1 indicates total pessimism.

Table 4 shows the finite element analysis results after applying the Hurwicz multi-criteria decision on the Pareto solution set based on optimistic, critical, and pessimistic attitudes. Compared with the initial design, all the performance indicators of the three decision designs improved. Among them, *CV*(*i_n_*)_a_ for the optimistic decision design decreased by 54.08%, response time *t*_0.95_ for the critical decision design decreased by 15.65%, and *a* for the pessimistic decision design increased by 10.32%.

## 6. Conclusions

(1) There exists a pronounced contradiction between the output electromagnetic force *F*_0.95_ and the response time *t*_0.95_, as indicated by a correlation coefficient of 0.859. Similarly, a significant contradiction is observed between the output electromagnetic force *F*_0.95_ and *CV*(*i_n_*), with a correlation coefficient ranging from 0.293 to 0.603. Moreover, a robust coupling relationship is discernible among *CV*(2), *CV*(3), and *CV*(4), as evidenced by a correlation coefficient spanning from 0.436 to 0.864.
(2) *α* has the most significant positive main effect on *CV*(*i_n_*). *N* and *L*_3_ have obvious negative main effects on *CV*(*i_n_*). The main effect of *α* and *d*_1_ on the output electromagnetic force *F*_0.95_ and response time *t*_0.95_ is strong, and the influence directions are the same. This indicates that the optimization of the long-stroke MPSA presents a robust nonlinear challenge.
(3) The MLP and RBF models are recommended for constructing the multi-objective optimization mathematical model of the long-stroke MPSA. Its optimal solution is solved using NSGA-II and determined using the Hurwicz criterion. The results show that *CV*(*i_n_*)_a_ for the optimistic decision design decreased by 54.08%, response time *t*_0.95_ for the critical decision design decreased by 15.65%, and *a* for the pessimistic decision design increased by 10.32%.

## Figures and Tables

**Table 1:** Key design parameters.

Parameters	Range
cone angle *α* (degree)	3–15
cone length *L*_1_ (mm)	20–32
inner hole depth of coil skeleton *L*_3_ (mm)	0–6
coil turns *N*	450–750
cone width *d*_1_ (mm)	1.25–7.25
cone inner diameter *r*_1_ (mm)	10–16
armature initial position *x*_0_ (mm)	−4–8

**Table 2:** Correlation strengths.

Correlation Strength	Absolute Values of Correlation Coefficient
low	0–0.3
medium	0.3–0.6
high	0.6–1

**Table 3:** The best electromagnetic force surrogate models at different operating points.

*F*(*i_n_*, *x_m_*)	*F*(*i_n_*,5)	*F*(*i_n_*,6)	*F*(*i_n_*,7)	*F*(*i_n_*,8)
*i_n_* (A)				
2	MLP	MLP	MLP	MLP
3	KR	KR	RBF	RBF
4	RBF	RBF	RBF	RBF
*F*(*i_n_*, *x_m_*)	*F*(*i_n_*,9)	*F*(*i_n_*,10)	*F*(*i_n_*,11)	*F*(*i_n_*,12)
*i_n_* (A)				
2	RBF	MLP	RBF	RBF
3	RBF	RBF	RBF	RBF
4	RBF	KR	KR	RBF
*F*(*i_n_*, *x_m_*)	*F*(*i_n_*,13)	*F*(*i_n_*,14)	*F*(*i_n_*,15)	-
*i_n_* (A)				
2	MLP	MLP	MLP	-
3	RBF	RBF	RBF	-
4	RBF	RBF	RBF	-

**Table 4:** Results of Hurwicz multi-criteria decision-making.

Design Parameters		*α*/deg	*L*_1_/mm	*L*_3_/mm		*N*	*d*_1_/mm		*r*_1_/mm	*x*_0_/mm
initial		8.88	24	0		590	4.25		11	2
optimized	optimistic decision (*λ* = 0)	8.92	20.00	4.25		671	4.65		10.00	1.43
	critical decision (*λ* = 0.5)	8.64	20.01	3.09		561	4.46		10.02	1.60
	pessimistic decision (*λ* = 1)	8.91	20.00	3.18		628	4.39		10.00	2.03
**Performance Index**		***CV*(*i_n_*)_a_**			***t*_0.95_/ms**			***a*/(m/s^2^)**		
initial		0.098			0.115			240.28		
optimized	optimistic decision (*λ* = 0)	0.045			0.113			260.45		
	critical decision (*λ* = 0.5)	0.064			0.097			255.56		
	pessimistic decision (*λ* = 1)	0.055			0.106			265.08		
rate of change (%)	optimistic decision (*λ* = 0)	−54.08			−1.74			8.39		
	critical decision (*λ* = 0.5)	−34.69			−15.65			6.36		
	pessimistic decision (*λ* = 1)	−43.88			−7.83			10.32		

## Data Availability

Important data are contained within the article. Additional data may be available upon reasonable request to the corresponding author. The data are not publicly available due to institutional policies.
